# The Royal Sanitary Institute

**Published:** 1918-07-27

**Authors:** 


### The Royal Sanitary Institute.

### LECTURES AND EXAMINATIONS FOR DISCHARGED MEN.

We are asked to state that the following concessions on account of war service have been made to discharged men by the Royal Sanitary Institute :—

Men discharged from the Navy or Army on account of wounds, disease, or other disablement contracted during naval or military service, are allowed to enter for the Institute's course of lectures, and for the Institute's examinations at half-fees, provided that their disability is not such as to prevent them from carrying out the duties of the office to which the examination applies. Candidates must have had the equivalent of practical training required of the regulations, and be able to either walk or bicycle five or six miles a day in order to get about a district. They must be able to go up a ladder; to use a note-book out of doors, write reports, and make entries in office books, and possess fairly good sight and sense of hearing and smell.

No great physical strength is required, and provided a man can comply with the above conditions an artificial limb or the loss of one eye would not in itself, we understand, debar him from taking up the work.

Sailors' or soldiers' widows whose husbands have been killed or have died during the present war, or sailors' or soldiers' wives whose husbands have been permanently disabled during the present war, are admitted to the courses of lectures, or the examinations of the Institute, at two-thirds of the ordinary fees.

